# A Quasi-Solid-State Polymer Lithium–Metal Battery with Minimal Excess Lithium, Ultrathin Separator, and High-Mass Loading NMC811 Cathode

**DOI:** 10.1021/acsaem.4c02099

**Published:** 2024-10-31

**Authors:** Gerrit Homann, Qing Wang, Sufu Liu, Antoine Devincenti, Pranav Karanth, Mark Weijers, Fokko M. Mulder, Matiss Piesins, Tom Gouveia, Alix Ladam, Sebastien Fantini, Corsin Battaglia

**Affiliations:** † Empa−Swiss Federal Laboratories for Materials Science and Technology, Überlandstrasse 129, 8600 Dübendorf, Switzerland; ‡ Delft University of Technology, Chemical Engineering Department, Van der Maasweg 9, Delft 2629HZ, The Netherlands; § Sidrabe Vacuum, Krustpils Iela 17, Riga 1073, Latvia; ∥ Solvionic, 11 Chemin des Silos, Toulouse 31100, France; ⊥ ETH Zurich, Department of Information Technology and Electrical Engineering, Gloriastrasse 35, 8092 Zürich, Switzerland; # EPFL, School of Engineering, Institute of Materials, Station 7, 1015 Lausanne, Switzerland

**Keywords:** solid-state batteries, polymer, polymerized ionic liquid, thin lithium, high-mass-loading NMC811, infiltration

## Abstract

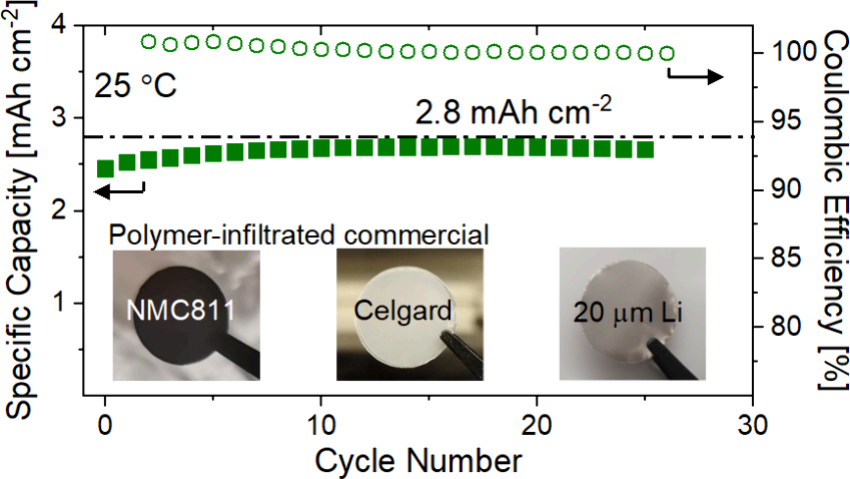

Solid-state batteries with lithium metal anodes are considered the next major technology leap with respect to today’s lithium-ion batteries, as they promise a significant increase in energy density. Expectations for solid-state batteries from the automotive and aviation sectors are high, but their implementation in industrial production remains challenging. Here, we report a solid-state lithium–metal battery enabled by a polymer electrolyte consisting of a poly(DMADAFSI) cationic polymer and LiFSI in Pyr_13_FSI as plasticizer. The polymer electrolyte is infiltrated and solidified in the pores of a commercial LiNi_0.8_Mn_0.1_Co_0.1_O_2_ (NMC811) cathode with up to 2.8 mAh cm^–2^ nominal areal capacity and in the pores of a 25 μm thin commercial polypropylene separator. Cathode and separator are finally laminated into a cell in combination with a commercial 20 μm thin lithium metal anode. Our demonstration of a solid-state polymer battery cycling at full nominal capacity employing exclusively commercially available components available at industrial scale represents a critical step forward toward the commercialization of a competitive all-solid-state battery technology.

## Introduction

Solid-state batteries employing solid electrolytes are projected to reach energy densities of >400 Wh kg^–1^ and >1200 Wh L^–1^, enabling long-distance electric road vehicles and short-haul electric aircrafts, respectively.^1−5^ Achieving such high energy densities is possible by combining a lithium metal anode (3860 mAh g^–1^, 3.04 V vs Li^+^/Li^0^) and a nickel-rich layered oxide cathode, such as LiNi_0.8_Mn_0.1_Co_0.1_O_2_ (NMC811, 200 mAh g^–1^, when cycled to >4.3 V vs Li^+^/Li^0^). Essential for the operation of such a battery is a suitable solid electrolyte that not only possesses sufficiently high lithium-ion conductivity and low gravimetric density but also offers compatibility with the lithium anode and sufficient oxidative stability.^6−8^

We recently identified the polymer poly(diallyldimethylammonium) bis(fluorosulfonyl)imide (PDADMAFSI) and *N*-butyl-*N*-methylpyrrolidinium bis(trifluoromethylsulfonyl)imide (Pyr_13_FSI) as a plasticizer in combination with lithium bis(fluorosulfonyl)imide (LiFSI) as a lithium salt as a promising electrolyte for solid-state batteries compatible with lithium metal anodes and NMC811 cathodes, demonstrating an excellent capacity retention of 72% after 600 cycles to 4.4 V at 25 °C.^9^ With a room-temperature ion conductivity of ∼1 mS cm^–2^, a gravimetric density of only 1.6 g cm^–3^, and a projected cost of $80 kg^–1^ at 50 t annual production, this nonflammable polymer electrolyte is a strong contender for a competitive solid-state battery technology.

However, most solid-state lithium metal cells reported in the literature (including ours in ref (9)) make use of prohibitively thick lithium metal anodes (>200 μm), to counter lithium inventory depletion during cycling, excessively thick separators (>100 μm for polymer electrolytes and often up to 1 mm thick for inorganic solid electrolytes such as sulfides, halides, oxides, and hydroborates^9−12^), due to processing-related issues and/or to prevent/delay short circuiting of cells by lithium metal dendrites, and cathodes with relatively low areal capacity (∼1 mAh cm^–2^), due to kinetic limitations and/or chemomechanical issues during discharge/charge cycling.^2,4,13−17^ To reach high energy density, it is of paramount importance to minimize the lithium metal anode thickness (also important for safety and cost), reduce the thickness of the separator between anode and cathode to a few tens of micrometers, and increase the areal capacity of the cathode to commercially viable values.^18^ These aspects are often neglected in solid-state cells reported in the scientific literature but are key for reaching high energy density and moving toward industrially relevant solid-state battery prototypes.

Here we demonstrate processes that enable the fabrication of solid-state lithium–metal battery cells exclusively from commercially available components with an only 20 μm thick lithium metal anode, an infiltrated industry-standard, 25 μm thin, porous polypropylene separator, and an infiltrated industrially manufactured NMC811 cathode with areal capacities up to 2.8 mAh cm^–2^ providing a realistic blueprint for the manufacturing of competitive solid-state batteries at scale.

## Experimental Section

**Figure 1:**
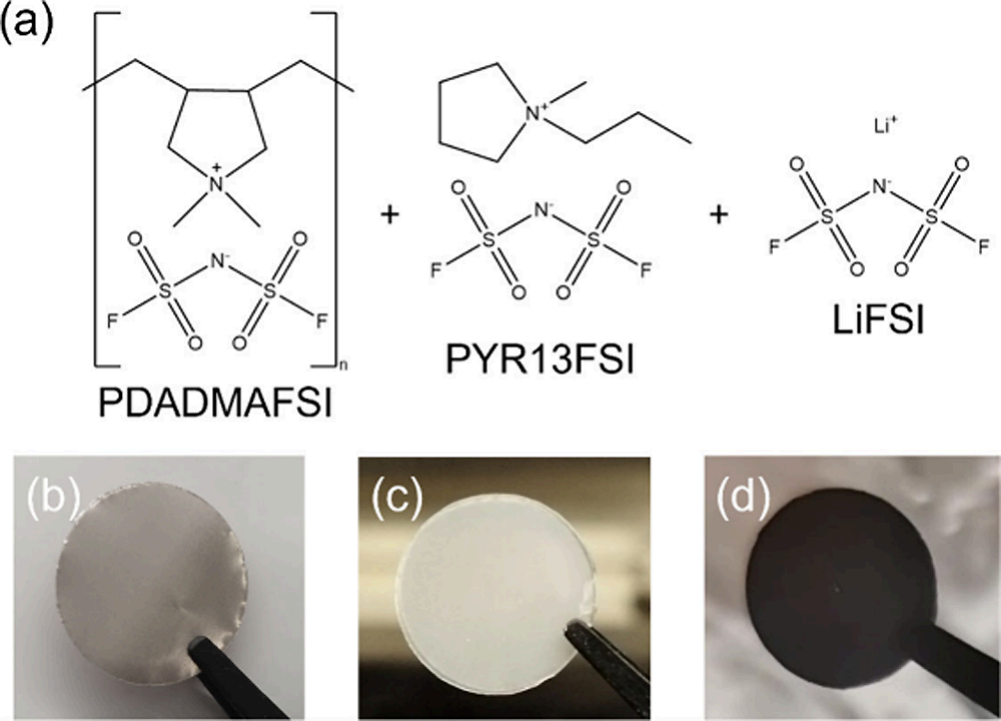
(a) Chemical structure of the components of the polymerized-ionic-liquid-based polymer electrolyte and photos of (b) a 20 μm thin lithium anode on a copper current collector, (c) an infiltrated 25 μm thick propylene separator, and (d) an infiltrated NMC811 electrode with a nominal areal capacity of 2.8 mAh cm^–2^.

Lithium metal was evaporated thermally in the form of a 20 μm thick uniform layer onto a 10 μm thick Cu current collector foil in an industrial roll-to-roll evaporation system under vacuum (<2 × 10^–5^ mbar) (Sidrabe Vacuum Ltd.). 25 μm thick porous polypropylene separators (Celgard 2500, pore size 64 nm, porosity 55%) and 260 μm thick glass fiber separators (Whatman GF/A, pore size 1.6 μm, porosity 90%) were punched into discs with a diameter of 16 mm, dried in vacuum (<10^–3^ mbar) for 12 h, and soaked in a solution containing PDADMAFSI polymer (40 wt %) and 1 or 3 M LiFSI (purity 99.9%) in Pyr_13_FSI (60 wt %) as plasticizer (see Figure 1a) in acetonitrile (electrolyte-to-solvent ratio 1:1 by weight) (Solvionic) for 12 h, dried passively in a polytetrafluoroethylene (PTFE) dish, soaked for an additional 1 h, dried at 55 °C in vacuum (<10^–3^ mbar) for 12 h, and transferred into an argon-filled glovebox (see Figure 1b). Commercial NMC811 electrodes with a nominal capacity of 1.0 mAh cm^–2^ (mass loading 6.3 mg cm^–2^) (Custom Cells) and 2.8 mAh cm^–2^ (mass loading 16.1 mg cm^–2^) (Lifun) were fixed on a glass plate and blade-coated with a solution containing PDADMAFSI polymer (40 wt %) and 1 or 3 M LiFSI (purity 99.9%) in Pyr_13_FSI (60 wt %) as plasticizer in propylene carbonate (electrolyte-to-solvent ratio 1:1 by weight) (Solvionic) using a blade-to-electrode gap of 100 μm. The high solubility of PDADMAFSI in propylene carbonate and the low viscosity of propylene carbonate are critical for the infiltration of the polymer electrolyte into the pores of thick NMC811 electrodes. Electrodes were subsequently dried at room temperature for 6 h in vacuum (<10^–3^ mbar) in the antechamber of an argon-filled glovebox. The low volatility of propylene carbonate prevents skin formation near the electrode surface during this step. Coating and drying were repeated and electrodes were then punched into discs with 12 mm diameter and dried at 55 °C in vacuum (<10^–3^ mbar) for 12 h, and transferred into an argon-filled glovebox (see Figure 1c). Cells were assembled using two-electrode coin cells (MTI R2032) by stacking either two lithium metal electrodes (Li||Li cells) separated by an infiltrated separator or a lithium metal electrode and an infiltrated NMC811 electrode (NMC811||Li) separated by an infiltrated separator. Mild stack pressure (estimated <0.2 MPa) is applied employing a 1.5 mm thick stainless steel spacer disc with a diameter of 15 mm and a stainless steel spring with a height of 1.4 mm. Galvanostatic cycling was conducted on multichannel potentiostats (Li||Li cells on Biologic MPG2 and NMC811||Li on Biologic BCS) in a climate chamber set to 25 °C. The total ion conductivities of the polymer electrolytes were measured using electrochemical impedance spectroscopy conducted from 1 MHz to 1 Hz with a voltage amplitude of 10 mV (Novocontrol). Lithium-ion transference numbers *t*_Li^+^_ were determined using the Bruce–Vincent method subjecting the cells to a polarization of 10 mV and measuring the current response for 12 h.^19^ Details concerning X-ray photoelectron spectroscopy (Thermo Scientific K-Alpha) and nuclear magnetic resonance (Bruker Ascend 500 MHz) measurements are provided in the Supporting Information.

## Results and Discussion

### Minimal Excess Lithium Enabled by Enhanced Coulombic Efficiency

**Figure 2:**
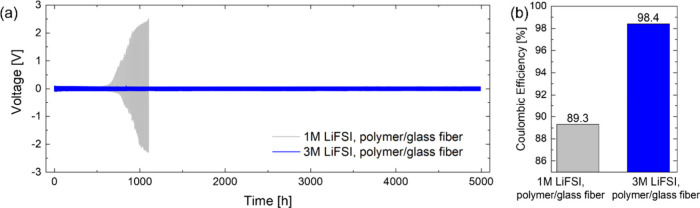
Lithium plating and stripping experiments in symmetric Li||Li cells at 25 °C. Comparison of (a) cell voltage and (b) average Coulombic efficiency between polymer electrolytes with 1 and 3 M LiFSI at 0.1 mA cm^–2^ and 0.1 mAh cm^–2^ per half cycle.

For safety and cost reasons, it is imperative to move to solid-state batteries with a minimum of excess lithium. However, reducing the thickness of the lithium metal anode represents a formidable challenge because thick lithium masks and compensates lithium inventory losses during cycling. This is illustrated in Figure 2a (gray curve), where the voltage of a symmetric Li||Li cell consisting of two 20 μm thick lithium electrodes separated by a glass fiber separator infiltrated by the polymer electrolyte with 1 M LiFSI cycled at a current density of 0.1 mA cm^–2^ and with 0.1 mAh cm^–2^ transferred per half cycle starts to diverge already after 600 h, leading to cell failure shortly after 1000 h. For comparison, an analogous cell with 250 μm thick lithium electrodes, shown in Figure S1, cycles stably for more than 1200 h with the cell voltage remaining below 100 mV. This is clear evidence that the consumption of lithium during cycling, caused by a low Coulombic efficiency, represents the main failure mode in the cell with thin lithium electrodes.

**Figure 3:**
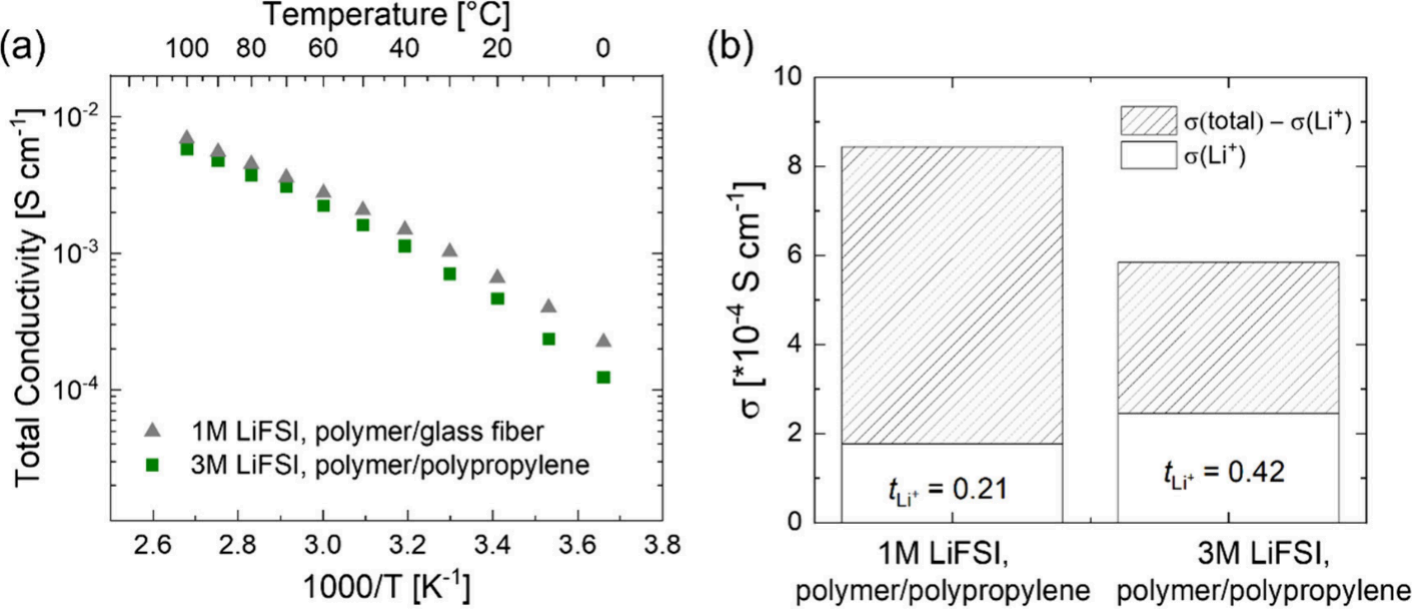
Comparison of polymer electrolyte with 1 and 3 M LiFSI in terms of (a) total ion conductivity vs temperature and (b) total ion vs lithium-ion conductivity at 25 °C. Because the polymer electrolyte was infiltrated into a polypropylene separator (see Ultrathin Commercial Separator That Also Benefits Coulombic Efficiency), measured effective conductivity values were scaled by a factor equal to the quotient of the polypropylene separator tortuosity of 2.5 and porosity of 0.55,^23,24^ resulting in conductivity values consistent with the values reported by some of us for freestanding polymer electrolyte sheets in ref (9).

Increasing the salt concentration has been widely accepted as a viable approach to improve the Coulombic efficiency of lithium anodes in liquid-electrolyte batteries.^20−22^ Inspired by this approach, we adopted a similar strategy for the polymer electrolyte. As a compromise between ion concentration and ion mobility, we increased the salt concentration from 1 to 3 M. As a consequence, the total ion conductivity of the polymer electrolyte at 25 °C decreases from 8.4 × 10^–4^ to 5.8 × 10^–4^ S cm^–1^ as shown in Figure 3. In contrast, the lithium-ion transference number at 25 °C increases from 0.21 to 0.42,^9^ resulting in an overall increase in the lithium-ion conductivity from 1.8 × 10^–4^ to 2.5 × 10^–4^ S cm^–1^ as a consequence of the increased salt concentration as visualized in Figure 3b.

Moreover, the increase in LiFSI salt concentration leads to a remarkable improvement in cycling stability of the symmetric Li||Li cell with 20 μm thick lithium electrodes, shown by the blue curve in Figure 2a, achieving stable cycling for more than 5000 h. The overpotential remains below 100 mV even after a cumulative charge transfer of 500 mAh cm^–2^. This value largely exceeds the capacity of the 20 μm thick lithium reservoir layer provided at the start, corresponding to only 4 mAh cm^–2^.

The average Coulombic efficiency of lithium plating and stripping is determined according to eq 6 proposed by Adams et al.^25^ with 4 mAh cm^–2^ of excess lithium (*Q*_T_ in ref (25), followed by repeated plating and stripping of 0.1 mAh cm^–2^ per half cycle (*Q*_C_ in ref (25) for 600 h for the cell with 250 μm thick lithium (before the cell voltage diverges) and 5000 h for the cell with only 20 μm thick lithium (providing a lower bound for the average Coulombic efficiency, as the cell keeps cycling beyond the 5000 h). Following this analysis, the Coulombic efficiency, graphically summarized in Figure 2b, improves from 89.3% to 98.4% when increasing the LiFSI salt concentration in the polymer electrolyte from 1 to 3 M, highlighting the much improved stability of lithium plating and stripping.

**Figure 4:**
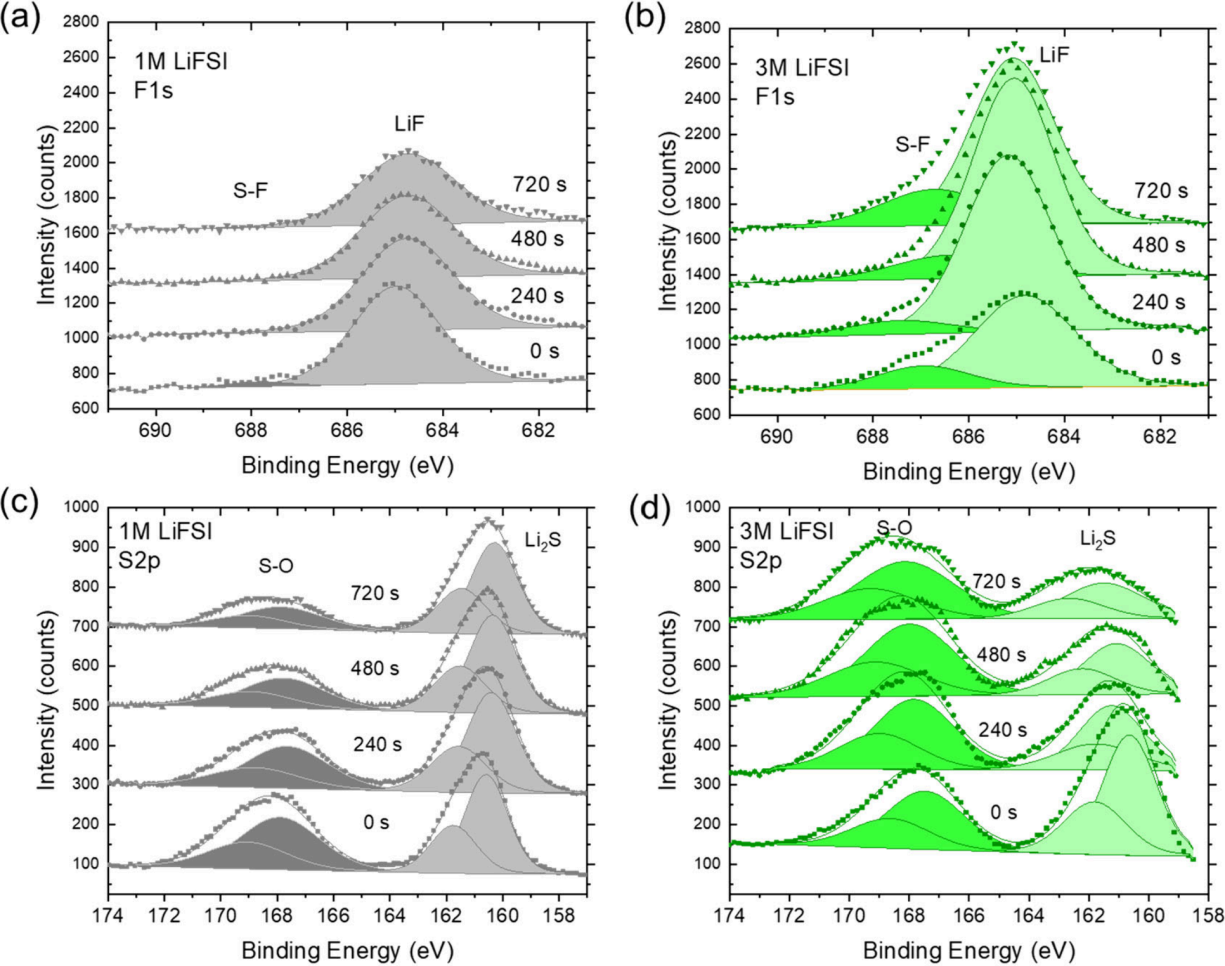
X-ray photoelectron spectra of the solid electrolyte interphase forming on the lithium metal anode in contact with polymer electrolyte with (a,c) 1 M LiFSI and (b,d) 3 M LiFSI.

To understand the improved Coulombic efficiency, we employed X-ray photoelectron spectroscopy (XPS) to study the composition of the solid electrolyte interphase forming between the lithium metal anode and the 1 or 3 M LiFSI polymer electrolyte. The XPS survey spectra and atomic composition of the SEI as a function of etch time are shown in Figure S2. The C 1s and O 1s spectra are shown in Figure S3. Figure 4a,b shows a comparison between the F 1s photoelectron spectra with a peak associated with LiF. While the F 1s peak intensity is comparable for both electrolytes directly after transfer into the ultrahigh vacuum chamber, the F 1s peak intensity increases rapidly as a function of argon-ion sputtering time for the 3 M LiFSI electrolyte, indicating that the bulk of its solid electrolyte interphase is LiF rich. Conversely, from comparison of the S 2p photoelectron peak intensities in Figure 4c,d, we conclude that the Li_2_S content in the solid electrolyte interphase from the 3 M polymer electrolyte decreases from the surface toward the bulk, while the 1 M polymer electrolyte results in a solid electrolyte interphase with a relatively homogeneous composition depth profile.

This trend is consolidated by magic-angle-spinning nuclear magnetic resonance spectroscopy, where a more intense peak corresponding to LiF is observed at a chemical shift of −205 ppm for the polymer electrolyte with 3 M LiFSI (Figure S4). Altogether, both XPS and NMR results indicate a solid electrolyte interphase richer in LiF in the case of the 3 M LiFSI containing polymer electrolyte, which improves the Coulombic efficiency. Ultimately it is not only the compositional profile and balance between LiF and other lithiated species but also its nanostructure that defines how a solid electrolyte interphase performs in the cell.^26^

### Ultrathin Commercial Separator That Also Benefits Coulombic Efficiency

As previously shown for cells with liquid electrolyte, the type of separator used can influence the morphology of the plated lithium.^27^ Replacing the freestanding polymer electrolyte separator by a 260 μm thick glass fiber separator infiltrated with the polymer also proved effective in our previous study in delaying dendrite formation thanks to enhanced mechanical properties and tortuosity.^9^ However, reducing the separator thickness further is crucial to achieving competitive energy density at the cell level. We have thus selected a 25 μm thin porous polypropylene separator, commercially available as Celgard 2500, as a candidate for this purpose.

Moreover, in order to pair thin lithium anodes with cathodes having commercially viable areal capacity, it is important to prevent lithium metal dendrite formation, which becomes more acute when a larger amount of charge is transferred per half cycle. In this study, we choose to increase the areal capacity transferred per half cycle from 0.1 mAh cm^–2^ to 2 mAh cm^–2^ while maintaining the current density at 0.1 mA cm^–2^. The 2 mAh cm^–2^ corresponds to a representative minimal threshold for the areal cathode capacity of a commercially viable battery technology.

**Figure 5:**
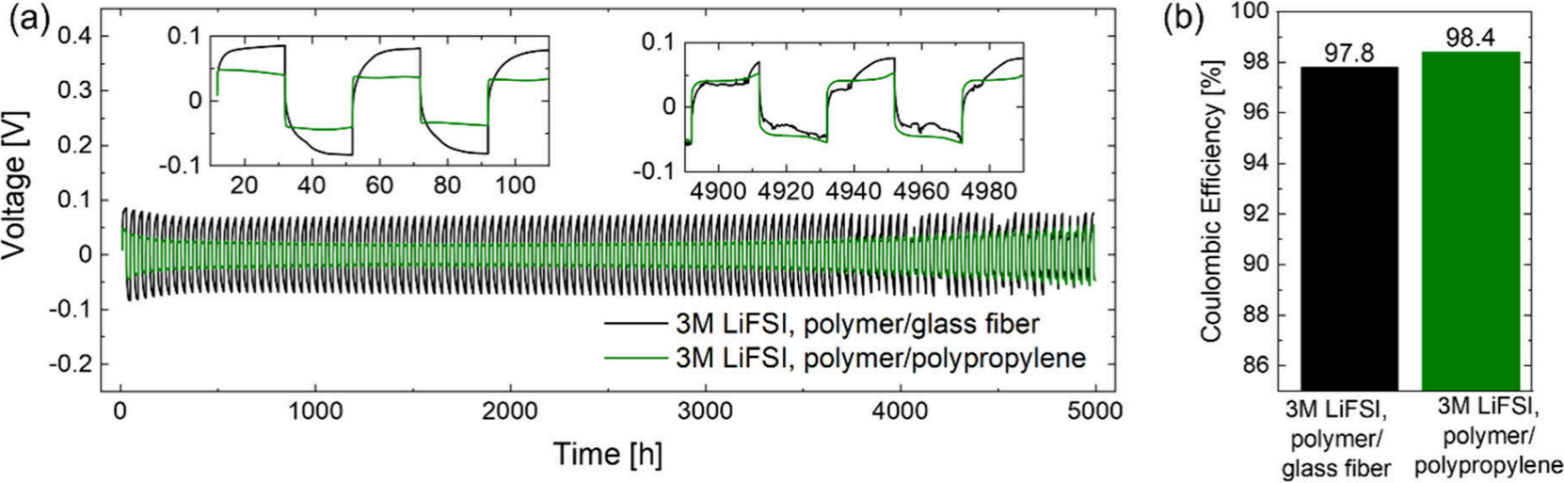
Lithium plating and stripping experiments of symmetric Li||Li cells at 25 °C. Comparison of (a) cell voltage and (b) average Coulombic efficiency between infiltrated glass fiber and infiltrated polypropylene separator infiltrated with 3 M LiFSI containing polymer electrolyte at 0.1 mA cm^–2^ and 2.0 mAh cm^–2^ per half cycle.

Figure 5a compares the cycling results with 2 mAh cm^–2^ transferred per half cycle for symmetric Li||Li cells with the 260 μm thick polymer-electrolyte-infiltrated glass fiber separator and a much thinner, only 25 μm thick, polymer-electrolyte-infiltrated polypropylene separator. As can be seen from inspection of Figure 5a, remarkably, the 25 μm thin infiltrated polypropylene separator is also capable of suppressing dendrite formation for more than 5000 h and results in an even more stable and overall significantly lower cell voltage during cycling. As shown in Figure 5b, the average Coulombic efficiency calculated according to ref (25) over 5000 h of plating and stripping reaches 97.8% for the 260 μm thick infiltrated glass fiber separator and 98.4% for the 25 μm thin infiltrated polypropylene separator. We emphasize again that these values represent merely a lower bound to the Coulombic efficiency, as the cells do not fail after 5000 h.

On inspecting the insets in Figure 5a, a clear difference in the shape of the voltage profiles is observed. The arcing voltage profile observed for the glass fiber separator can be related to mass transport limitations caused by the accumulation of electrolyte decomposition products, e.g. dead lithium or detached solid electrolyte interphase fragments, effectively increasing the tortuosity of lithium-ion diffusion at the interface.^28,29^ In contrast, the cell with the polypropylene separator shows a flatter voltage profile, which suggests denser, but mossy lithium forming between anode and electrolyte as observed also in scanning electron microscopy images after cycling and disassembly of our cells (Figure S5).^28,30^ Altogether, the cycling stability in Li||Li cells is clearly improved with the polypropylene separator, probably due to the smaller average pore size of the polypropylene separator (64 nm) in comparison to the glass fiber separator (1.6 μm), promoting uniform plating and stripping. As a consequence of the infiltration process, both separators exhibit a thin layer of excess polymer electrolyte on the top and bottom surfaces after soaking, preventing direct contact with the lithium metal.

### High-Mass-Loading NMC811

**Figure 6:**
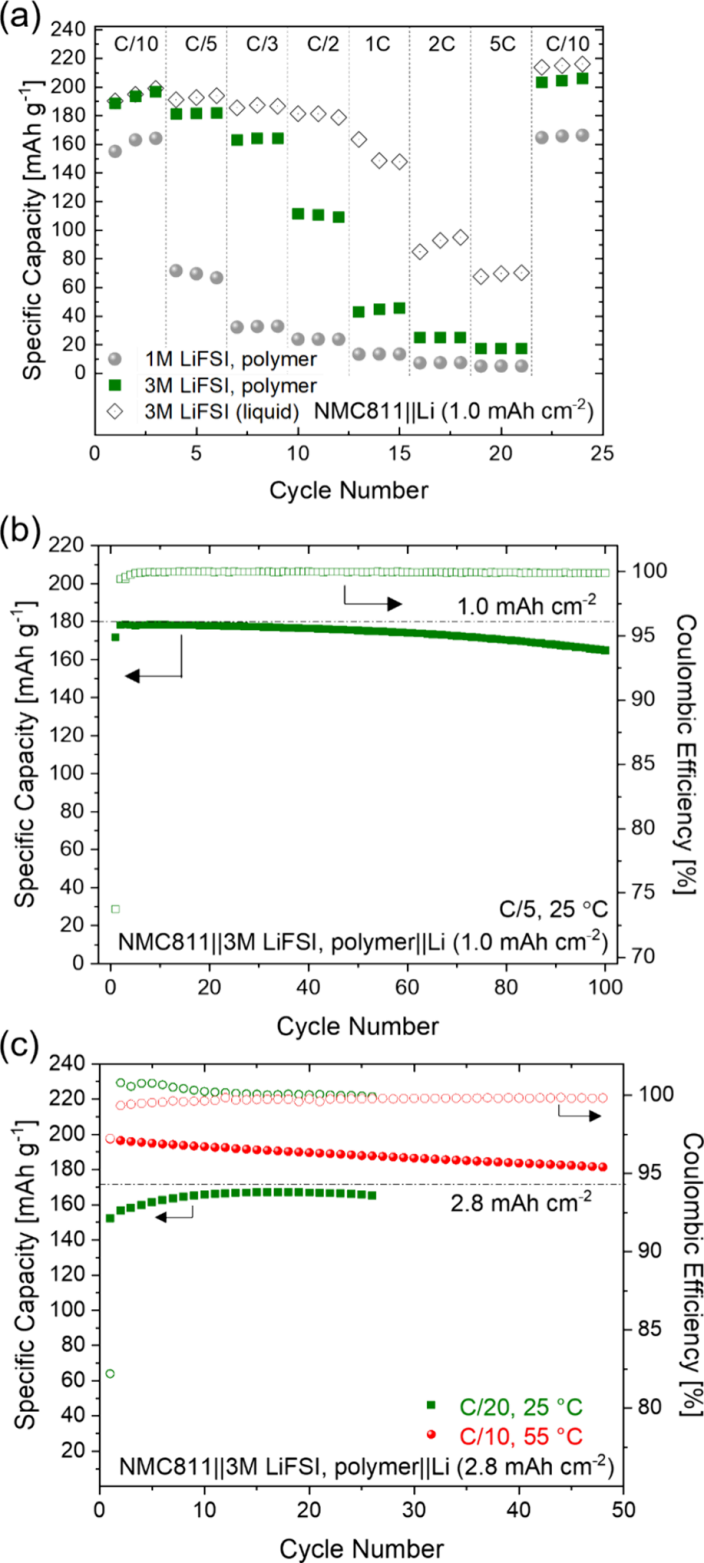
Cycling performance of NMC811||Li full cells cycled from 3.0 to 4.4 V. (a) Galvanostatic C-rate experiment at 25 °C comparing cells with 1 and 3 M LiFSI containing polymer electrolyte against a cell with a benchmark 3 M LiFSI in Pyr_13_FSI liquid electrolyte. The polymer electrolyte was infiltrated into the NMC811 cathode and the Celgard separator, while a glass fiber separator was used for the cell with liquid electrolyte due to the poor wettability of Pyr_13_FSI on polypropylene. Long-term galvanostatic charge/discharge cycling of the cell with 3 M LiFSI containing polymer electrolyte at (b) C/5 with 1 mAh cm^–2^ cathode at 25 °C and (c) C/20 with 2.8 mAh cm^–2^ NMC811 cathode at 25 and 55 °C. Dashed lines indicate nominal capacity values provided by the electrode suppliers.

We continue to demonstrate the attractiveness of the infiltration process in enabling NMC811||Li full cells by infiltrating our polymer electrolyte into commercial NMC811 electrodes (for process details, see the Experimental Section). When pairing the thin lithium metal anode and the thin infiltrated polypropylene separator with an NMC811 cathode with 1 mAh cm^–2^ areal loading, the cell with the 3 M LiFSI containing polymer electrolyte exhibits significant enhancement in rate capability at 25 °C compared to the cell with 1 M LIFSI, especially at rates exceeding C/5 as shown in Figure 6a. The cell with the 3 M LiFSI containing polymer delivers a capacity of up to 200 mAh g^–1^ when charged at C/10 to 4.4 V vs Li^+^/Li^0^, which is as good as the capacity achieved using a liquid reference electrolyte consisting of 3 M LiFSI in Pyr_13_FSI. This suggests excellent contact between the polymer electrolyte and the NMC811 cathode particles achieved by infiltration. It is interesting to note that the contact seems to improve further over time, resulting in even higher capacities in the C/10 recovery cycles (cycles 22–24). As expected, the rate capability of the cells with the polymer electrolyte remains lower than that of the reference cell with the liquid electrolyte.

The evolution of the discharge capacity and Coulombic efficiency of NMC811||Li full cells with a 1.0 mAh cm^–2^ cathode during long-term galvanostatic cycling at C/5 is shown in Figure 6b. After a formation cycle with a low Coulombic efficiency of 74%, the average Coulombic efficiency over the subsequent 100 cycles is 99.92%, resulting in an excellent capacity retention of 92% after 100 cycles. With a capacity of 178 mAh g^–1^, this cell also reaches the full nominal capacity of 1.0 mAh cm^–2^ specified by the electrode supplier (indicated by the dashed horizontal line).

In Figure 6c we demonstrate that the infiltration process can also be applied successfully to an NMC811 cathode with a high, commercially relevant areal capacity of 2.8 mAh cm^–2^. After 15 cycles at C/20, during which the contact between the cathode and the infiltrated electrolyte continues to improve, the discharge capacity reaches its maximum at 174 mAh g^–1^, which reaches the nominal capacity of 2.8 mAh cm^–2^ specified by the electrode supplier (indicated by the dashed horizontal line). While increasing the discharge rate capability of this cell remains a challenge at 25 °C, higher rates can be reached at 55 °C (Figure S6). Higher capacities of up to 200 mAh g^–1^ can be reached at C/10 with decent cycling stability at 55 °C, as also shown in Figure 6c.

The combination of a 20 μm lithium metal anode on a 10 μm Cu foil with a 25 μm thick infiltrated polypropylene separator and a 2.8 mAh cm^–2^ infiltrated NMC811 cathode is projected to deliver an energy density on cell level >360 Wh kg^–1^ (see calculation in the Supporting Information). Energy densities ∼400 Wh kg^–1^ are projected to be achievable with the same combination of components increasing the areal capacity of the cathode to 4.5 mAh cm^–2^, which remains challenging but nevertheless a reasonable target. Alternatively, an energy density of ∼430 Wh kg^–1^ can also be reached for a lower areal capacity of the cathode of 2.8 mAh cm^–2^ by eliminating the copper current collector and using the lithium metal anode as a current collector. Future efforts also have to focus on enabling such high areal capacities to be cycled at higher rates.

## Conclusion

In conclusion, we have demonstrated enhanced Coulombic efficiency by increasing the LiFSI concentration from 1 to 3 M in a polymerized ionic liquid electrolyte, which results in a more robust and LiF-richer solid electrolyte interphase as confirmed by XPS and NMR. We also showed the attractiveness of infiltrating the polymer into commercial polypropylene separators and high-mass-loading NMC811 electrodes, thereby enabling decent performance with light weight and simplified processing. Our proof-of-concept study shows that solid-state batteries incorporating lithium metal anodes and NMC811 cathodes with industrially relevant areal capacity can be assembled from components that are all commercially available at a scale that enables the transfer to a 50 MWh y^–1^ pilot solid-state battery manufacturing line.

While thin lithium metal anodes on copper foils, polypropylene separators, and NMC811 electrodes are already available from a number of commercial suppliers at market prices, the polymer electrolyte is projected to become available at volumes of 50 t y^–1^, matching the 50 MWh y^–1^ cell production, at a cost of $80 kg^–1^.^9^

Addressing and solving manufacturing challenges at scale are key for solid-state batteries to be adopted in battery gigafactories in the near future. In this respect, the infiltration and solidification of polymer electrolytes into commercial separators and commercial NMC811 cathodes represent additional steps in the manufacturing process. However, they can be integrated into existing separator and cathode manufacturing and cell assembly already established in battery gigafactories, thereby minimizing adoption barriers and financial risks when transitioning from the manufacturing of traditional lithium-ion batteries to the manufacturing of next-generation solid-state batteries.

## Supporting Information Available

Additional details on methods, supporting electrochemistry, XPS, NMR figures and SEM images, and calculation of projected energy densities on cell level (PDF)
