# Supplementary material for: A Quasi-Solid-State Polymer Lithium–Metal Battery with Minimal Excess Lithium, Ultrathin Separator, and High-Mass Loading NMC811 Cathode
Source: ACS Appl Energy Mater. 2024 Oct 31;7(21):10037–43. doi: 10.1021/acsaem.4c02099 (PMC11558555; doi:10.1021/acsaem.4c02099)
Supplement: Supplementary file 1 — ae4c02099_si_001.pdf [file ae4c02099_si_001.pdf]

Supporting Information

A quasi-solid-state polymer lithium-metal battery with minimal excess lithium, ultrathin separator, and high-mass loading NMC811 cathode

Gerrit Homann,[a,*,‡] Qing Wang,[a, ‡] Sufu Liu,[a] Antoine Devincenti,[a] Pranav Karanth,[b] Mark Weijers,[b] Fokko M. Mulder,[b] Matiss Piesins,[c] Tom Gouveia,[d] Alix Ladam,[d] Sebastien Fantini,[d] Corsin Battaglia[a,e,f]

[a]*Empa – Swiss Federal Laboratories for Materials Science and Technology, Überlandstrasse 129, 8600 Dübendorf, Switzerland*

[b]*Delft University of Technology, Chemical Engineering Department, Van der Maasweg 9, Delft 2629HZ, The Netherlands*

[c]*Sidrabe Vacuum, Krustpils Iela 17, Riga 1073, Latvia*

[d]*Solvionic, 11 Chemin des Silos, Toulouse 31100, France*

[e]*ETH Zurich, Department of Information Technology and Electrical Engineering, Gloriastrasse 35, 8092 Zürich, Switzerland*

[f]*EPFL, School of Engineering, Institute of Materials, Station 7, 1015 Lausanne, Switzerland*

* gerrit.homann@empa.ch

Additional details on methods, supporting electrochemistry, XPS, NMR figures and SEM images, and calculation of projected energy densities on cell level.

Magic-angle-spinning nuclear magnetic resonance (MAS NMR) spectroscopy

To obtain solid electrolyte interphase samples for MAS solid-state NMR measurements, Li||Cu coin cells containing the Celgard separator with the 1M/3M LiFSI polymer electrolyte were cycled for 20 cycles (Maccor 4300, 0.1 mA cm^{-2}, 0.5 mAh cm^{-2} plating/stripping per half cycle with 30 min rest), and subsequently the Cu foils containing the Li deposits were extracted from these cells. The deposited Li layer plus its solid electrolyte interphase was then scraped off from the Cu foils (unwashed) and was packed into 3.2 mm airtight ZrO_2 rotors with KBr as the inert filler.

All solid state NMR measurements were carried out on a Bruker Ascend 500 MHz (11.7 T) spectrometer equipped with a Neo console. ^{7}Li, ^{19}F, and ^{1}H MAS NMR measurements were carried out on a 3.2 mm triple resonance probe at spinning speeds of 18 kHz using direct excitation pulses. For ^{7}Li measurements, a pulse length of 5 μs (45 W) was used with a recycle delay of 30 s. All measurements were referenced to a LiCl solution at 0 ppm. ^{19}F measurements were carried out with a pulse length of 3.25 μs (107.75 W) and recycle delay of 10 s. ^{1}H measurements were carried out with a pulse length of 3.25 μs (107.75 W) and recycle delay of 4 s. These measurements were referenced to adamantane ^{13}C frequency at 1.75 ppm. The processing and analysis of the resulting spectra was performed using the Mestrenova 11 software.

X-ray photoelectron spectroscopy (XPS)

XPS measurements with depth profiling were also carried out on lithium deposited on copper foils, collected from Li||Cu cells as described above. Before XPS measurements, the foils were washed with dimethyl carbonate (Sigma Aldrich, 99%) to remove LiFSI salt deposits and dried in a glovebox antechamber overnight at room temperature. Instead of scraping off the Li, the washed Cu foils were directly used for the analysis. The samples were transferred to the XPS chamber using a vacuum transfer chamber.

The XPS measurements were performed on a Thermo Scientific K-Alpha Spectrometer with an Al K-alpha monochromator. The spot diameter was set to 400 μm. Survey spectra were recorded with a pass energy of 200 eV and step size of 0.5 eV, while high-resolution spectra were recorded with a pass energy of 50 eV and step size of 0.1 eV. The binding energies were referenced to adventitious carbon at 284.8 eV. For the depth profiling measurements, argon ion sputtering was performed (1 keV ion energy,

1.5 μA and 2x2 mm^2 raster size). All the spectra were processed and analyzed using the CasaXPS software using a U2 Tougaard background to account for inelastically scattered photoelectrons.

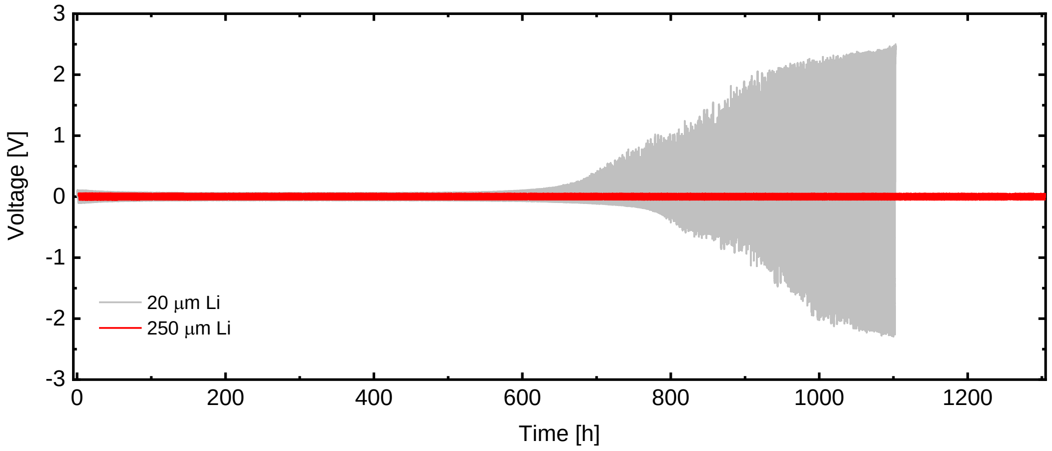

Figure S1: Comparison of lithium plating and stripping in symmetric Li||Li cells with thin lithium metal (20 μm, 4 mAh cm^{-2}, grey curve) and thick lithium metal (250 μm, 50 mAh cm^{-2}, red curve) at 0.1 mA cm^{-1}, 0.1 mAh cm^{-2} per half cycle, and 25 °C. Interestingly, the cycle life of the cells is varying with the amount of lithium metal excess, pointing towards the consumption of active lithium as the major mode of failure for the 1M LiFSI containing polymer electro-lyte.

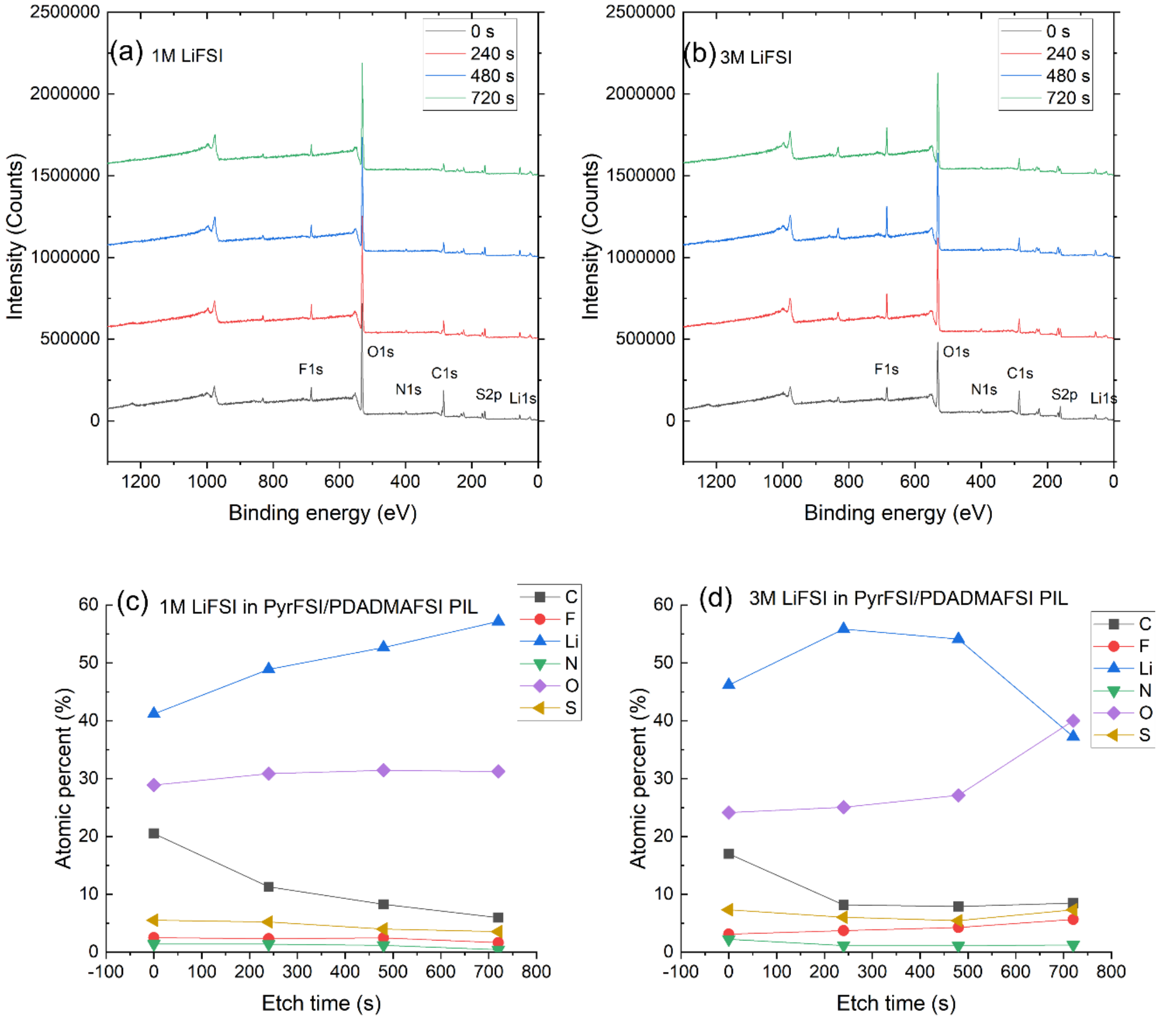

Figure S2: XPS survey spectra and elemental percentages at different etch time of the solid electrolyte interphase forming on the lithium metal anode in contact with polymer electrolyte with (a,c) 1M LiFSI and (b,d) 3M LiFSI.

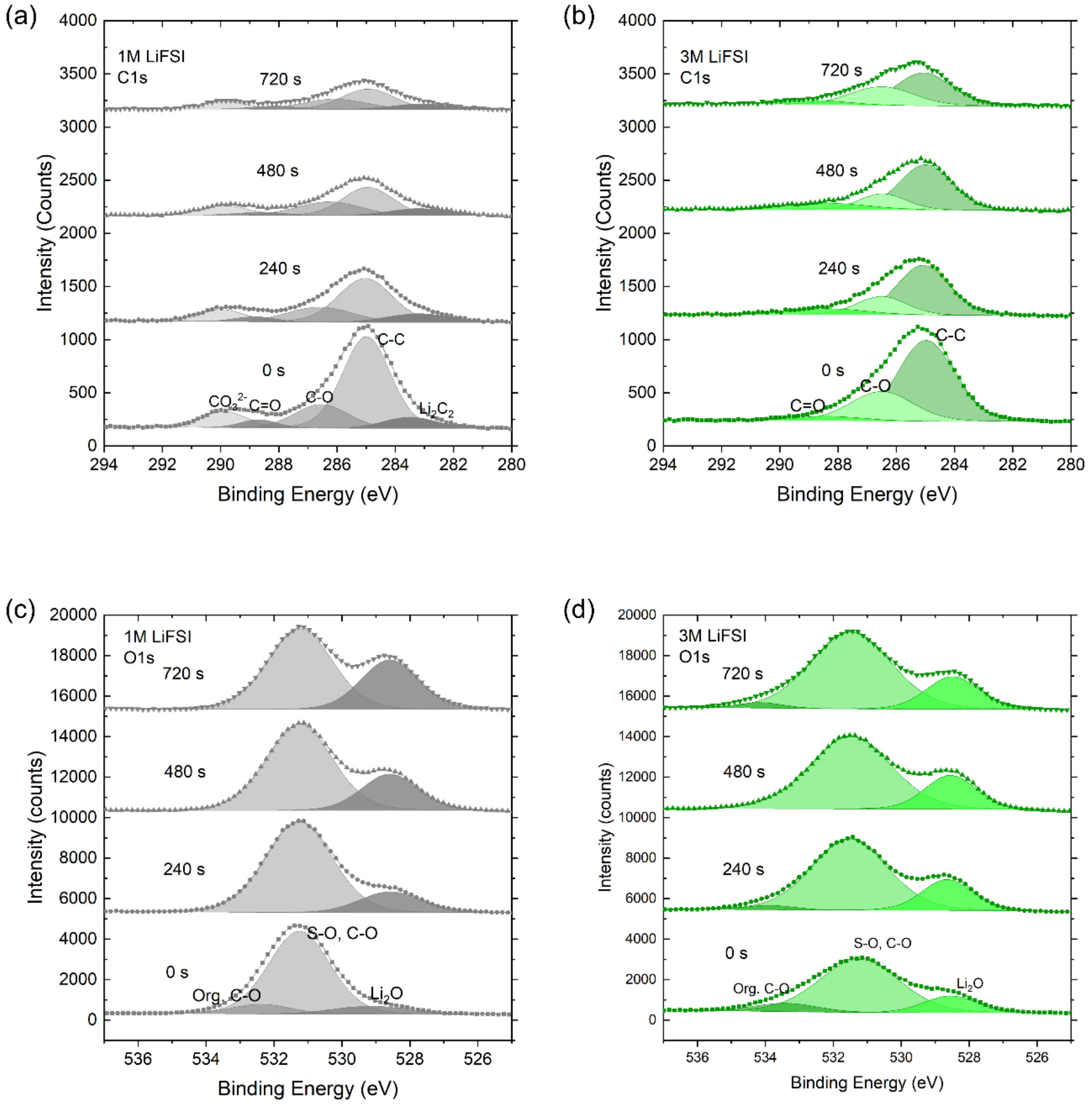

Figure S3: C1s and O1s XPS spectra of the solid electrolyte interphase forming on the lithium metal anode in contact with polymer electrolyte with (a,c) 1M LiFSI and (b,d) 3M LiFSI.

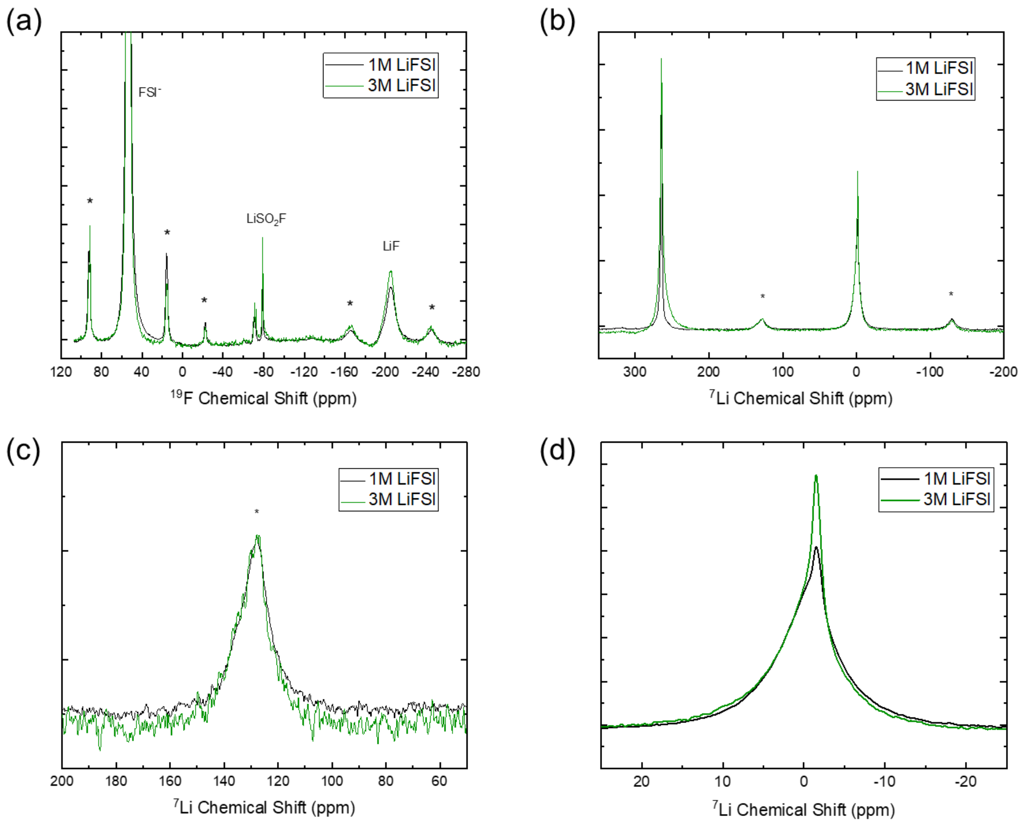

Figure S4: Magic-angle-spinning nuclear magnetic resonance spectra of the solid electrolyte interphase forming between the lithium metal anode and the polymer electrolyte with 1M LiFSI and 3M LiFSI. a) ^{19}F spectra with spinning sidebands denoted by asterisks. The peak at 52 ppm corresponds to LiFSI, the one at 205 ppm corresponds to LiF. The feature at 79 ppm was previously attributed to $LiSO_2F$,[26] a decomposition product known to occur in FSI-based electrolytes. A higher LiF content is observed in the solid electrolyte interphase of the polymer electrolyte with 3M LiFSI consistent with the XPS analysis in Figure 4. b) ^{7}Li overview spectra with c) zoom into the spinning sideband at 128 ppm, which were used for normalizing the spectra, and d) zoom into the diamagnetic region containing signatures from the LiFSI salt and solid electrolyte interphase environments.

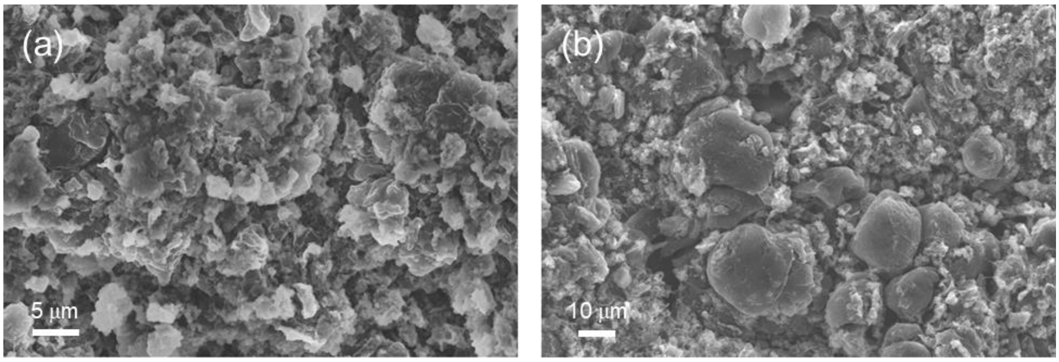

Figure S5: Scanning electron microscopy images of lithium metal anode after 100 h lithium plating and stripping (50 cycles, 10 mAh cm^{-2}, at 0.1 mA cm^{-1}, 0.1 mAh cm^{-2} per half cycle, and 25 °C) in symmetric Li||Li cell with polymer electrolyte with a) 1M LiFSI and b) 3M LiFSI. The lithium metal surface was washed with propylene carbonate and ethyl methyl carbonate to remove polymer residues before transfer into the electron microscopy. The images indicate that the increased LiFSI concentration results in denser deposition of lithium on the surface of the lithium electrode.

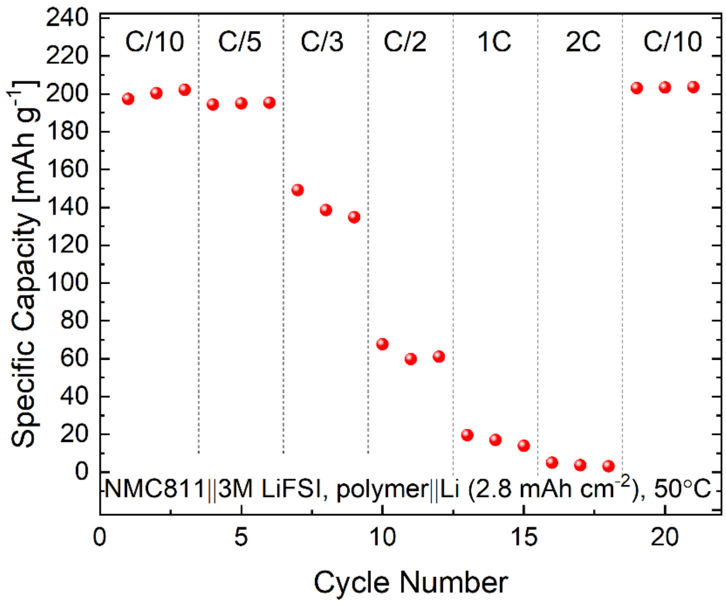

Figure S6: Galvanostatic C-rate experiment at 55 °C of 3M LiFSI containing polymer electrolyte with 2.8 mAh cm^{-2} NMC811 cathode.

Calculation of projected energy density on cell level

For the calculation of the energy density, the following values were used

Average discharge voltage	3.85 V
Discharge capacity	2.8 mAh cm^{-2}
Average discharge energy	3.85 V * 2.8 mAh cm^{-2} = 10.78 mWh cm^{-2}

Density Li metal	0.534 g cm^{-3}
Thickness Li metal	20 μm
Weight Li metal	0.534 g cm^{-3} * 20 μm = 1.1 mg cm^{-2}
Density polypropylene	0.9 g cm^{-3}
Thickness separator	25 μm
Porosity separator	55%
Weight separator	0.9 g cm^{-3} * 25 μm * (100% - 55%) = 1.0 mg cm^{-2}
Thickness cathode	46 μm
Porosity cathode	30%
Cathode active material content	96.4%
Weight cathode	16.1 mg cm^{-2} / 97% = 16.7 mg cm^{-2}
Density polymer electrolyte	1.6 g cm^{-3} (1.6-1.7 g cm^{-3} depending on solvent and drying conditions)
Weight electrolyte in separator	1.6 g cm^{-3} * 25 μm * 55% = 2.2 mg cm^{-2}
Weight electrolyte in cathode	1.6 g cm^{-3} * 46 μm * 30% = 2.2 mg cm^{-2}
Density Cu	8.96 g cm^{-3}
Thickness Cu current collector	10 μm
Weight Cu current collector	8.96 g cm^{-3} * 10 μm = 9.0 mg cm^{-2}
Density Al	2.70 g cm^{-3}
Thickness Al current collector	10 μm
Weight Al current collector	2.70 g cm^{-3} * 10 μm = 2.7 mg cm^{-2}

The total cell weight is calculated by summing the weights of all components but taking into account only half of the Cu and Al current collector weight, because we assume in a commercial cell the current collectors will be double side coated. In addition, we are neglecting the weight of the packaging foil/can of the cell, which is an assumption asymptotically valid for increasing cell size.

Total cell weight	29.6 mg cm^{-2} (without rounding weight of every component)
Average discharge energy	3.85 V * 2.8 mAh cm^{-2} = 10.78 mWh cm^{-2}
Energy density	10.78 mWh cm^{-2} / 29.6mg cm^{-2} = 364 Wh kg^{-1}

Analogous calculation but assuming a cathode with 4.5 mAh cm^{-2} and 100 μm thickness.

Weight cathode	26.8 mg cm^{-2} / 97% = 27.6 mg cm^{-2}
Weight electrolyte in cathode	1.6 g cm^{-3} * 100 μm * 30% = 4.8 mg cm^{-2}
Total cell weight	42.62 mg cm^{-2} (without rounding weight of every component)
Average discharge energy	3.85 V * 4.5 mAh cm^{-2} = 17.33 mWh cm^{-2}
Energy density	17.33 mWh cm^{-2} / 42.62 mg cm^{-2} = 407 Wh kg^{-1}

Analogous calculation but assuming a cathode with 2.8 mAh cm^{-2} and 46 μm thickness and no copper current collector.

Total cell weight	25.1 mg cm^{-2} (without rounding weight of every component)
Average discharge energy	3.85 V * 2.8 mAh cm^{-2} = 10.78 mWh cm^{-2}
Energy density	10.78 mWh cm^{-2} / 25.1 mg cm^{-2} = 430 Wh kg^{-1}
